# Particularly neglected in countries with other challenges: High *Toxoplasma gondii* seroprevalence in pregnant women in Kabul, Afghanistan, while a low proportion know about the parasite

**DOI:** 10.1371/journal.pone.0223585

**Published:** 2019-10-10

**Authors:** Sayed Hussain Mosawi, Zahra Zarghona, Abdolhossein Dalimi, Pikka Jokelainen, Ahmad Hosseini Safa, Mohammad Reza Mohammadi, Erfan Javanmardi, Mohammad Baqer Basirat

**Affiliations:** 1 Medical Sciences Research Center, Ghalib University, Kabul, Afghanistan; 2 Faculty of Medicine, Khatam Al Nabieen University, Ghazni, Afghanistan; 3 Department of Infectious Diseases, Afghanistan National Charity Organization for Special Diseases, Kabul, Afghanistan; 4 Faculty of Medicine, Khatam Al Nabieen University, Kabul, Afghanistan; 5 Department of Medical Parasitology, Faculty of Medical Sciences, Tarbiat Modares University, Tehran, Iran; 6 Laboratory of Parasitology, Department of Bacteria, Parasites & Fungi, Infectious Disease Preparedness, Statens Serum Institut, Copenhagen, Denmark; 7 Department of Medical Parasitology and Mycology, School of Public Health, Tehran University of Medical Sciences, Tehran, Iran; 8 Medical Laboratory Department, French Medical Institute for Children, Kabul, Afghanistan; Jazan University, SAUDI ARABIA

## Abstract

Toxoplasmosis is a zoonotic parasitic disease of global importance. It is widespread and endemic practically all over the world, with infection prevalence varying by geographic location. The parasite and the disease are neglected, which is illustrated by the lack of baseline information on the prevalence from many regions. Developed, peaceful regions are in better situation to address toxoplasmosis, while the neglected status is particularly pronounced in regions with other challenges. Due to the lack of baseline data, these regions are rarely mentioned in discussions about the neglected status of the disease. A dramatic manifestation of toxoplasmosis, congenital toxoplasmosis, is the dreaded outcome of vertical transmission of the infection from the mother to the unborn child. For this reason, pregnant women are a key target group for primary prevention of *T*. *gondii* infections, and baseline data on the prevalence in pregnant women is important. In this cross-sectional seroepidemiological study, we estimated *Toxoplasma gondii* seroprevalence and evaluated possible risk factors for seropositivity in pregnant women in Kabul, Afghanistan. Altogether 207 of the 431 women included in the study tested positive for immunoglobulin G antibodies against *T*. *gondii*, yielding an apparent seroprevalence estimate of 48.03% (95% CI 43.33–52.75). Based on the final multivariable model for *T*. *gondii* seropositivity, non-concrete floor in the house and well or river as water source were identified as risk factors for seropositivity, while residence in rural area was a protective factor. The majority of the participants (72.9%) reported that they did not know about *T*. *gondii*. Our study is the first to report an estimate of *T*. *gondii* seroprevalence in pregnant women in Afghanistan. The high seroprevalence indicates substantial infection pressure, and the results of the risk factor analysis suggest that the environmental route, infection from oocysts, might be the most relevant to address to prevent the infections in the region. Our results contribute to the global discussion on neglected status of toxoplasmosis.

## Introduction

Toxoplasmosis is a zoonotic parasitic disease caused by an obligate intracellular protozoan parasite, *Toxoplasma gondii*. The parasite has a cosmopolitan distribution, and it has been estimated that about one third of the world’s population is infected [1]. Beside humans, the parasite can infect a vast variety of warm-blooded animals, and it has an environmentally resistant form, oocyst. The infection can be transmitted to humans by ingestion of sporulated oocysts, which have been shed in unsporulated form by infected felids (environmental route), or by ingestion of tissue cysts, which are present in tissues of infected animals (meatborne route). The tachyzoites of *T*. *gondii* have the ability to cross the placenta [2].

While most *T*. *gondii* infections are subclinical, the disease, toxoplasmosis, can be fatal [1]. Toxoplasmosis is a neglected disease of global importance. It is widespread and endemic practically all over the world, with prevalence varying by geographic location—and lack of baseline information from many regions [3,4].

Congenital toxoplasmosis results from vertical transmission of the infection from the mother to the unborn child and causes a substantial disease burden [2,3]. Congenital toxoplasmosis is approached very differently by region [5] and not at all in many regions.

Developed, peaceful regions are in better situation to address toxoplasmosis, while the neglected status is particularly pronounced in regions with other challenges. Such regions are often not even considered in the discussions about neglected status of congenital toxoplasmosis [5,6]. This is partly due to lack of studies from these regions, which thus remain without a voice.

Afghanistan, a country with a population of approximately 30 million, has been afflicted by series of armed conflicts. The seroprevalence of *T*. *gondii* infection has not been previously estimated for pregnant women in Afghanistan, and there is no systematic serological screening of pregnant women for the infection in the country. The number of disability-adjusted life years (DALYs), covering number of years lost and number of years lived with a disability, weighted reflecting the severity of the disability, due to congenital toxoplasmosis has been estimated to be 11,212 in the country [3]. However, it needs to be emphasized that due to lack of reported data for congenital toxoplasmosis and *T*. *gondii* seroprevalence, this estimate was modeled using data from neighboring countries. In this study, we addressed this data gap: we estimated *T*. *gondii* seroprevalence and evaluated possible risk factors for seropositivity in pregnant women in the capital city of Afghanistan, Kabul.

## Materials and methods

### Ethical considerations

For this seroprevalence study, ethical approval was obtained from Medical Ethic Committee of Research and Technology Center, Khatam Al Nabieen University, Ghazni, Afghanistan. All the protocols used in this study were in accordance with the approved guidelines (AF.GKNU.REC.1397.001). Participation was voluntary and all participants gave written informed consent. The data were handled confidentially and analyzed coded.

The observed outcome of the pregnancy of one participant is mentioned, in a way that it is not identifiable. We obtained oral informed consent for this over telephone discussion (ZZ); written informed consent was not possible to obtain due to geographical distances.

### Study design, setting and study population

This cross-sectional seroepidemiological study (S1 File) was conducted in 2017–2018 in three hospital and health centers in Kabul. Kabul is the capital and the largest city of Afghanistan, located in eastern part of the country, 1,790 meters above sea level. The study population comprised pregnant women who lived in Kabul area, who were registered as pregnant, and who underwent monthly follow-ups by obstetricians in the three hospital and health centers between February 2017 and April 2018. Participants fulfilling these inclusion criteria were recruited at the three hospital and health care centers.

No active follow-up of the participants was included in this study. Any results of any further testing and clinical follow-up fell outside the scope of this cross-sectional study.

### Sample size and sampling

We calculated the needed sample size using 95% confidence level, 5% confidence limits, and seroprevalence of *T*. *gondii* among pregnant women in northeastern Iran, 34.4% [7], as the expected seroprevalence. A minimum total sample size to obtain was 347.

The blood samples (5 ml) were collected at the three hospital and health care centers and transported to the Laboratory of French Medical Institute for Mothers and Children (FMIC), Kabul, Afghanistan. Serum was separated from whole blood by centrifugation and stored at -20°C until tested.

### Questionnaire

General socio-demographic data as well as history of exposure to potential risk factors were collected by interview using a structured questionnaire. The potential risk factors were selected based on the current knowledge of the life cycle and transmission routes of the parasite, focusing on food-borne (particularly meat-borne), water-borne, and soil-borne transmission. The questions covered age group, residence in urban or rural area, having university education or not, working or studying outside the home or not, having income level below or at least 10,000 Afghani, number of family members, owning cat or not, having a mainly vegetarian diet or meat-including diet, consuming raw milk or not, consuming raw meat or not, having central water source from pipe system or river or well as water source, boiling water before use or not, having concrete or non-concrete floor in the house, and having contact with soil for example in agricultural activities or in other work that is linked with soil or not (Table 1). Moreover, we surveyed whether the participants self-reportedly knew about *T*. *gondii* or toxoplasmosis or not. We did not collect any other medical data about the participants nor details of the pregnancies. The questions and answer options were simple, and we did not provide detailed definitions for the expressions used.

**Table 1:** Seroprevalence of *Toxoplasma gondii* in pregnant women in Kabul, Afghanistan.

Variable	N tested	n seropositive	% apparent seroprevalence	95% CI for the seroprevalence	Univariable odds ratio	95% CI for the odds ratio
Age group						
≤ 30 years	227	111	48.90	42.43–52.40		
> 30 years	204	96	47.06	40.27–53.93	0.93	0.64–1.36
Residential area						
Urban	343	175	51.02	45.73–56.29		
Rural	87	32	36.78	27.16–47.27	0.56*	0.34–0.91
University education						
Yes	18	7	38.39	18.86–62.25		
No	411	199	48.42	43.61–53.25	1.48	0.56–3.88
Work or study outside the home						
No	363	182	50.14	45.00–55.27		
Yes	67	24	35.82	25.05–47.80	0.56*	0.32–0.95
Income level						
< 10,000 Afghani	180	76	42.22	35.16–49.53		
≥ 10,000 Afghani	222	115	51.18	45.23–58.33	1.47	0.99–2.19
Family size						
≤ 4 family members	61	26	42.62	30.70–55.24		
> 4 family members	361	178	49.31	44.17–54.46	1.31	0.76–2.26
Owning cat						
No	262	121	46.18	40.20–52.25		
Yes	168	86	51.19	43.64–58.70	1.22	0.83–1.80
Diet						
Mainly vegetarian	126	60	47.62	39.00–56.34		
Including meat	302	146	48.34	42.74–53.98	1.03	0.68–1.56
Raw milk consumption						
No	410	195	47.56	42.75–52.40		
Yes	18	11	61.11	37.75–81.14	1.73	0.66–4.56
Raw meat consumption						
No	16	7	43.75	21.54–68.05		
Yes	413	198	47.94	43.15–52.77	1.18	0.43–3.24
Water source						
Central pipe system	166	63	37.95	30.81–45.51		
River or well	265	144	54.34	48.31–60.27	1.95*	1.31–2.89
Boiling of water before use						
Yes	6	2	33.33	6.02–73.81		
No	423	204	48.23	43.49–52.99	1.86	0.34–10.28
Floor type in the house						
Concrete	323	144	44.58	39.22–50.04		
Non-concrete	108	63	58.33	48.87–67.35	1.74*	1.12–2.70
Soil contact						
No	11	2	18.18	3.17–48.27		
Yes	419	205	48.93	44.16–53.71	4.31	0.92–20.19
Knowing about *Toxoplasma gondii*						
Yes	117	48	41.03	32.38–50.11		
No	314	159	50.64	45.11–56.15	1.47	0.96–2.27
Total	431	207	48.03	43.33–52.75		

Some questions were not answered by some participants.

The variables are presented so that the option coded ‘0’ is listed first.

CI = confidence interval

* = P value <0.05

### Serology

We used a semi-quantitative serological chemiluminescence immunoassay for detecting the presence of anti-*T*. *gondii* immunoglobulin G (IgG) antibodies in the samples, following the manufacturer’s instructions (IMMULITE^®^/IMMULITE^®^ 1000, Siemens, Eschborn, Germany). The sensitivity and specificity of the method have been reported to be 99% and 100%, respectively [8]. Each participant was categorized as either seronegative or seropositive based on the result from this single test: The result was considered positive if IgG antibody concentration was at least 8 international units (IU)/ml.

### Statistical analysis

OpenEpi was used for calculating 95% confidence intervals (CI, Mid-P Exact) for the prevalence estimates [9]. Seroprevalence that was adjusted for test sensitivity and specificity was estimated using EpiTools [10]. Stata IC 13.1 software (Stata Corporation, TX, USA) was used for the risk factor analyses. Questions had two answer options, with the exceptions of three age groups, four education levels, four categories for family size, three categories for income level, three options for meat in diet, and three options for water source. As these categorical variables did not appear to be statistically significantly associated with seropositivity (age group, education level, family size, income, meat in diet) or to provide additional value over dichotomized version of the variable (water source), they were dichotomized based on biological importance to allow simple comparisons. No answer was treated as missing data. After univariable (crude) analyses of the dichotomous and dichotomized variables, a multivariable regression model for seropositivity was built by including all the variables into the model, followed by stepwise elimination of variables with P-value >0.05 that did not act as confounders.

## Results

Altogether 431 pregnant women were included in the study. Of the 431 women, 207 tested seropositive, yielding an apparent seroprevalence estimate of 48.03% (95% CI 43.33–52.75). The seroprevalence that was adjusted for test sensitivity and specificity was 48.51% (95% CI 43.79–53.27). Table 1 shows the apparent seroprevalence by the variables. The majority of the participants (72.9%) reported that they did not know about *T*. *gondii*.

Despite we did not collect information about the pregnancies, outcomes and other tests performed, we were informed that one of the participants of this study gave birth to a child with macroscopic hydrocephalus that ruptured spontaneously during vaginal delivery. Reportedly, a medical doctor was consulted, and congenital toxoplasmosis was suspected but remained unconfirmed. The blood sample included in this study from the mother was positive for anti-*T*. *gondii* IgG antibodies (15.2 IU/ml). Evaluation of any further data on the pregnancy or her medical history and follow-up was beyond the scope of this study, but we were informed that this was her first pregnancy, and she had also tested positive for anti-*T*. *gondii* IgM (result 2.10; results >1.20 interpreted as positive), while serology results for Rubella, Cytomegalovirus and Herpes Simplex Virus were reportedly negative. We have no data on potential confirmation of the IgM result, timing of the testing, nor information on any further serological or clinical follow-up or treatment, but the outcome was reportedly unexpected for the mother. In our questionnaire, she reported consuming only well-cooked or well-washed food.

The final multivariable model for *T*. *gondii* seropositivity used 430 observations and had three variables: non-concrete floor in the house and well or river as water source were identified as risk factors for seropositivity, while residence in rural area was identified as a protective factor (Table 2).

**Table 2:** Variables of the final multivariable model for *Toxoplasma gondii* seropositivity in pregnant women in Kabul, Afghanistan.

Variable	Odds ratio	95% CI	P-value
Residence in rural area	0.38	0.23–0.66	<0.001
Non-concrete floor in the house	1.80	1.09–2.98	0.022
River or well as water source	1.94	1.26–2.97	0.002

Variables were dichotomous, the options were compared with the opposite (Table 1).

CI = confidence interval

## Discussion

Our study is the first to report an estimate of baseline *T*. *gondii* seroprevalence in pregnant women in Afghanistan. The estimate, 48.03%, is statistically significantly higher (P value 0.00005299) than the recent estimate from northeastern Iran 34.4% [7], which we used as the expected seroprevalence in the calculation of needed sample size. The high seroprevalence indicates substantial infection pressure.

The risk factors for *T*. *gondii* IgG seropositivity that we identified in this study indicate that the environmental oocyst reservoir would be the source of infection to address in this region. Eating raw meat did not appear as a risk factor for seropositivity in this study, whereas it has been identified as a risk factor in other studies [11]. It should however be emphasized, that the diet aspects were reported by the participants by answering simple questions, and more detailed data could be better for identifying possible foodborne risks. Moreover, only a small proportion of participants reported not consuming raw meat, and this result should thus be interpreted with caution. Non-concrete floor in the living house was identified as a risk factor in this study, as was using river or well as the water source. Interestingly, living in rural areas appeared as a protective factor, suggesting that the urban areas could perhaps have higher level of environmental contamination. These results appear contradicting and are thus interesting, but should be considered preliminary and interpreted with caution. Further studies should look into the details of potential exposure to oocysts from the environment, using more specific and direct questions, and investigate potential explanations for the observed difference between rural and urban areas.

Based on available literature, the seroprevalence of *T*. *gondii* infection has not been previously estimated for pregnant women in Afghanistan, and congenital toxoplasmosis has gained limited attention. Possibly linked to this, the majority of the participants reported that they had no previous knowledge about *T*. *gondii*. This question was simple and asked about knowing about the parasite or the disease, and could have been good to be supported by further questions asking e.g. about some specific manifestations, including any local words that there may be for them. On the other hand, detailed descriptions of the disease manifestations could have caused distress and worry in the participants. A simple, neutral question was selected for this study.

The results of our study must be interpreted cautiously, taking into account the many limitations of the study. It is important to emphasize that the aim of the study was to estimate the baseline seroprevalence and evaluate risk factors for IgG seropositivity in a relevant sub-population, pregnant women. Pregnancy was one of the inclusion criteria, but we did not collect medical data about the participants (e.g. trimester of pregnancy, medical history, other tests performed), because those data were not considered directly relevant for this study. Due to limited resources, it was also necessary to keep the study design simple. How well the convenience sample we investigated represents the whole population was not formally evaluated, and the results of this study should be generalized with caution and considered as the first, preliminary estimate. Our results can help in planning future studies, which should include more information about the participants, in particular obstetric data. For the aim of this study, this information was not collected because it was not considered important for the aim of the study (to estimate seroprevalence). However, it could have been useful for the usability of the results (science to clinical practice) and for evaluating potential sampling bias and referral bias. Moreover, some of those data could have been interesting for evaluating the importance of the parasite and congenital toxoplasmosis in the region, as well as to identify patterns to help target interventions.

Moreover, we focused only on IgG antibodies, which are the most suitable for epidemiological studies, and only one sample per participant was tested. Further results, for example, repeated testing, IgM result, IgG avidity data, direct detection of the parasite or its DNA (using PCR), and clinical follow-up data would be relevant for estimating the incidence of acute infections and congenital toxoplasmosis. A positive IgG results indicates earlier exposure but does not allow evaluating when the infection took place. Further studies, in particular studies with more clinical focus as well as larger epidemiological studies, are needed. This study, providing an estimate of baseline seroprevalence, can help in planning such future studies.

Overall, a major limitation of our study was the simplicity of the questions and evaluation of dichotomous or dichotomized variables, as well as that for some variables, the number of participants selecting a specific answer was small. Some questions were very general and could have been understood differently by some participants, while some were more clearly defined. The definitions were designed to fit the setting of this study, and some may not be comparable to similar variables in other studies.

The simple study design can also been seen as a strength. We were able to conduct this study in the difficult conditions, and this can empower research initiatives in other areas with challenges.

*Toxoplasma gondii* infections, which can have devastating consequences, affect people everywhere—also in regions with other challenges, such as Afghanistan. The high seroprevalence we report indicates high infection pressure, while the awareness appeared low. While we do not have all the information about the suspected case of congenital toxoplasmosis mentioned, it was an unexpected outcome for the mother. This may suggest suboptimal or lacking management of a possible case, that a possible maternal infection could have gone unnoticed despite at least one IgG and IgM result were available, or that communication to the mother could have failed. This situation happened and could be considered a third approach to congenital toxoplasmosis, in addition to the two diverging approaches described for France *vs*. the United States [5]. This illustrates inequity in health care across the world.

*Toxoplasma gondii* infections and congenital toxoplasmosis remain neglected even in developed, peaceful regions that would be able to address them appropriately [5,6], and the neglected status is even more pronounced in regions with other challenges. Lack of data hampers estimating [3] and addressing the burden the disease causes. This study is an example of a study filling in a baseline data gap and yielding data that can contribute to the discussion about the neglected status of toxoplasmosis in both local and global scales.

## Supporting information

S1 File STROBE checklist. (DOCX)
